# Invasive cystic hypersecretory carcinoma of the breast: a rare variant of breast cancer: a case report and review of the literature

**DOI:** 10.1186/s12885-018-5260-2

**Published:** 2019-01-08

**Authors:** Jie Sun, Xing Wang, Cuifang Wang

**Affiliations:** Department of Pathology, Central Hospital affiliated to Shenyang Medical College, No 5, South Seven West Road, Tiexi District, Shenyang, Liaoning 110024 People’s Republic of China

**Keywords:** Breast cancer, Cystic hypersecretory carcinoma, Cystic hypersecretory hyperplasia

## Abstract

**Background:**

Cystic hypersecretory carcinoma is a rare subtype of breast cancer. It is a member of cystic hypersecretory lesions, which include a series of pathological disease lineages: cystic hypersecretory hyperplasia (CHH), CHH with atypia, cystic hypersecretory carcinoma (CHC) and invasive CHC. It was found that most cystic hypersecretion lesions were in situ carcinoma, and only 19 cases of invasive cystic hypersecretion carcinoma were reported.

**Case presentation:**

We are reporting a case of a 63-year-old female who had a lump in her left breast for 3 years. A modified radical mastectomy was done and morphological diagnosis of invasive CHC with axillary node metastasis was made.

**Conclusions:**

Owing to a smaller number of reported cases, little is known about the biological behavior, prognosis and molecular study of cystic hypersecretion lesions. Therefore, more cases with follow-up data are needed to reveal the biological behavior of this rare tumor.

## Introduction

Cystic hypersecretory lesions of the breast have a spectrum of morphological features ranging from clearly benign (CHH), CHH with atypia, cystic hypersecretory carcinoma (CHC) to invasive CHC [1]. CHC was first described by Rosen PP and Scott M in 1984 [2]. The gross and microscopic features of this entity are unique. Gross detection shows numerous cysts of varying sizes. The contents of the cysts are gelatinous material. Microscopically, dilated ducts with marked secretory activity (the secretion is thyroid colloid–like substance) and lining by pseudostratified to micropapillary epithelium [1, 2]. Among the reported cases of cystic hypersecretory breast lesion, most cases are of in situ CHC, with only a few cases of invasive CHC. Invasion is suggested by solid nests structures and are usually poorly differentiated with no secretory characteristic. Extravasation of cyst material into the stroma does not indicate invasion [1, 2]. This rare subtype of breast cancer was not included in 2012 WHO Classification of Tumors of the Breast,but its unique gross and microscopic features triggered us to report this rare tumor. Until now, only 19 cases of invasive CHC have been reported in the literature [1–11]. Our case is the twentieth case and the relevant literature is reviewed (Table 1).

**Table 1:** Review of invasive CHCs in the literature

Soure, y	Age, y	Type of Disease Present	Lymph Node Status	ER/PR^a^
Rosen and Scott [2], 1984	52	Invasive	c	Pos/NA
	47	Invasive	N1	NA
	62	invasive	N0	NA
Guerry et al. [1], 1988	NA	Invasive	N1	NA
	NA	Invasive	c	NA
Adams and Lacey [12], 1990	70	Microinvasive	N0	Neg/pos
Kim et al. [3], 1997	37	Invasive	N0	NA
Herrmann et al. [4], 1999	49	Invasive	N0	Pos/pos
Lee JS [5], 2004	45	Invasive	N0	Neg/neg
Shin SJ [6], 2004	42^b^	Invasive	N (micro)	NA
Skalova A [7], 2005 (two cases)	66.8^b^	Invasive	NA	One Case Pos/pos
Chen DB [13], 2010	44	Microinvasive	NA	Pos/pos
Song SW [8], 2011	43	Invasive	NA	NA
D’Alfonso TM [14], 2014	62.8^b^	Microinvasive	NA	Pos/pos
Bi R [9], 2014	37	Invasive	N1	NA
	46	Invasive	NA	NA
Gupta P [10], 2014	57	Invasive	N0	Neg/neg
Sahoo N [11], 2017	32	Invasive	N1	Neg/neg
Present case	63	Invasive	N1	Neg/neg

^a^ER/PR indicates estrogen receptor/progesterone receptor; NA: not available;

pos: positive; neg: negative;

N (micro): lymph node micrometastasis

^b^Mean age

^c^Indicates cases with distal metastatic disease

## Case presentation

A 63-year-old female presented with a palpable mass in her left breast for 3 years. The lump was gradually progressive in size for the past 3 years. Physical examination revealed a painless, ill-defined, hard, large mass with no nipple discharge in the upper outer quadrant of the left breast. Skin dimpling and ulceration were also seen. The patient had no past or family history of a breast disease. A modified radical mastectomy was performed. The CAF chemotherapy was administered after surgery.

**Fig. 1:**
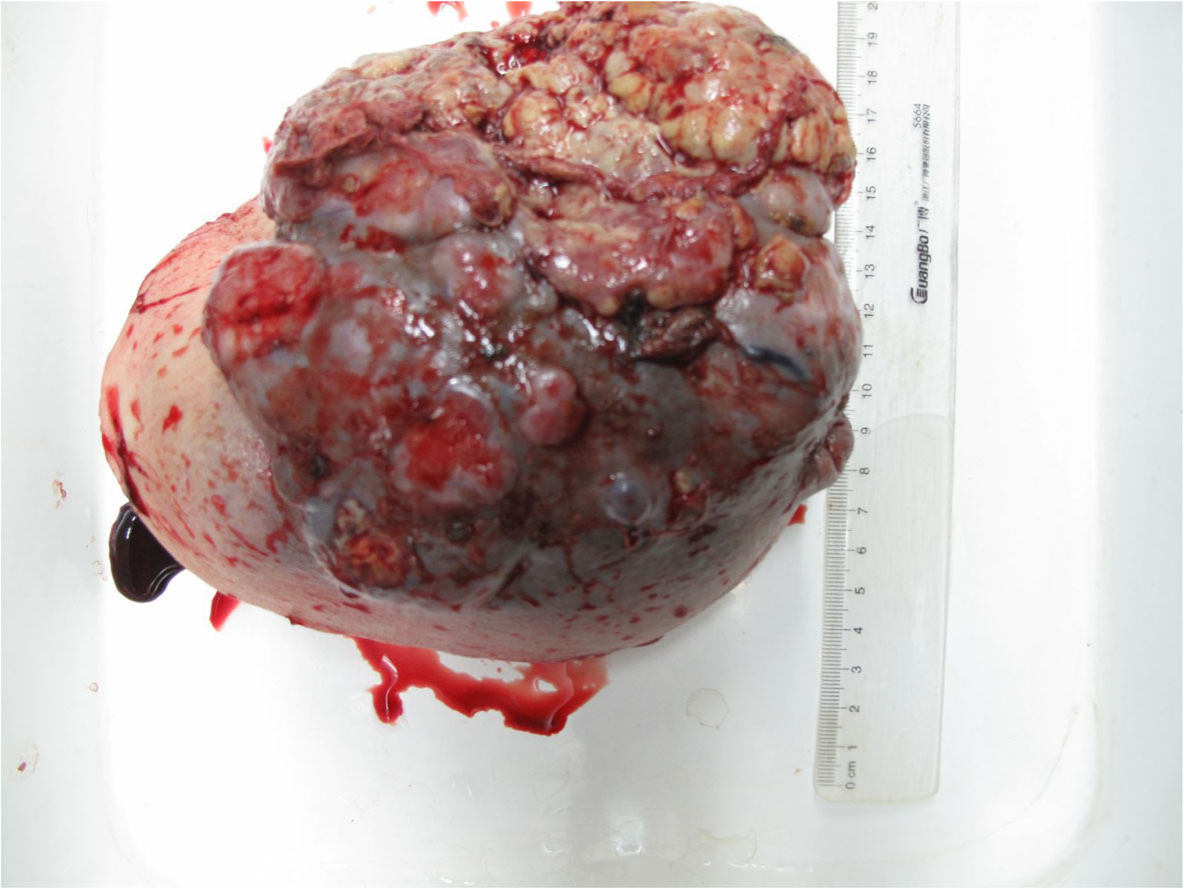
The surface of the mass showing an ill-defined, red gray, multiple nodular, 14 × 12 cm tumour with obvious skin ulceration

Grossly, the left breast specimen showed an ill-defined, red gray, multiple nodular, 14 × 12 cm tumor with surface skin ulceration [Fig. 1]. The cut surface revealed multiple cystic spaces filled with thick, gelatinous secretions and gray-white solid areas. The individual cysts varied from 0.2 cm to 2.5 cm in dimension with cysts wall thickness from 0.1 cm to 0.5 cm. Hemorrhage and necrosis was evident.

**Fig. 2:**
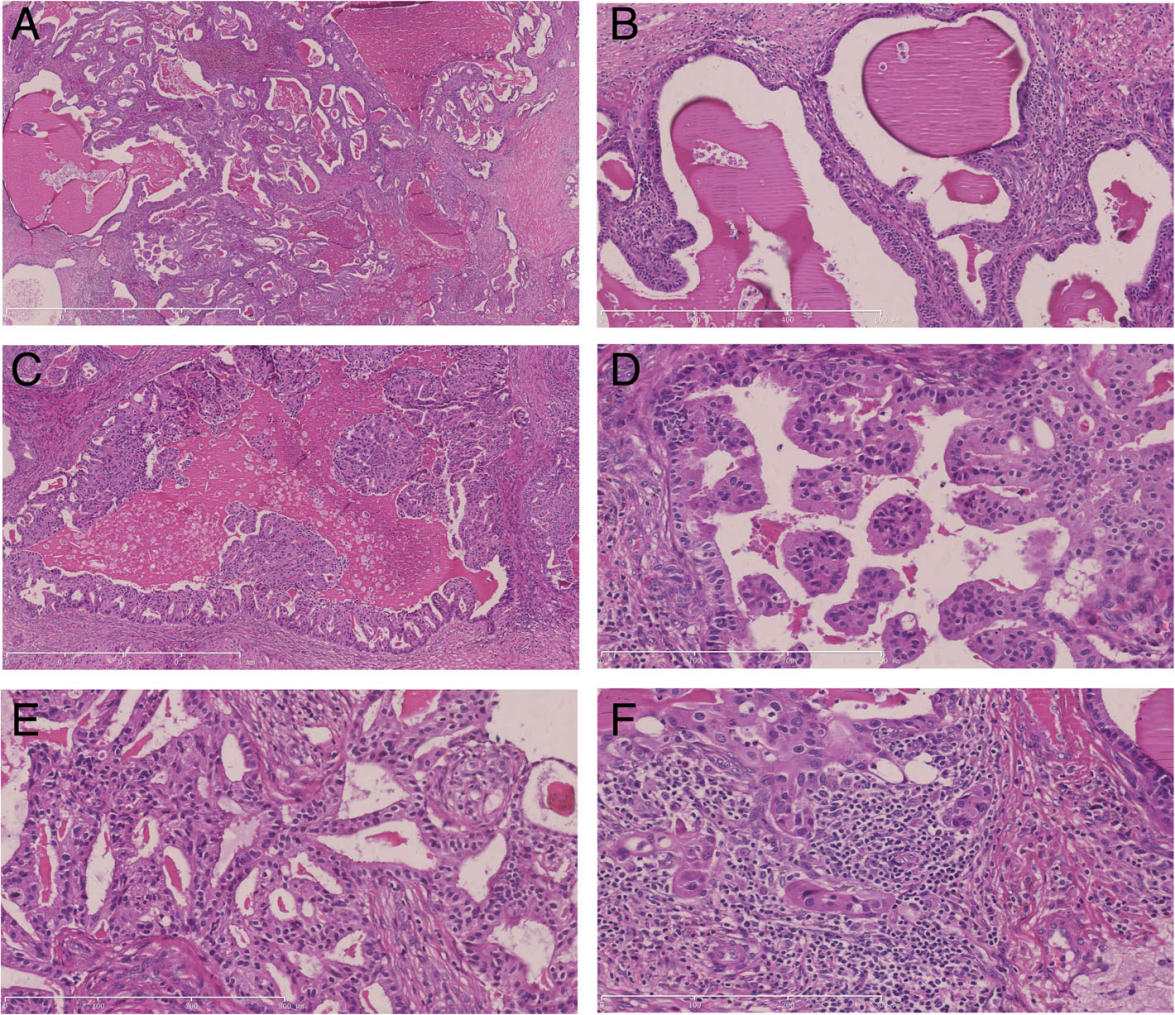
Microscopic findings. Multiple variable-sized cysts and ducts filled with thyroid colloid-like eosinophilic secretions (**a**, H&E, × 25). Some of the cysts are lined by flat to cuboidal epithelium (**b**, H&E, × 100). In other areas the epithelium showed a proliferative change in the form of pseudostratification, knobby tufts (**c**, H&E, × 50), micropapillary (**d**, H&E, × 200) and cribriform (**e**, H&E, × 100). An invasive component comprising of solid nests was seen (**f**, H&E, × 200)

**Fig. 3:**
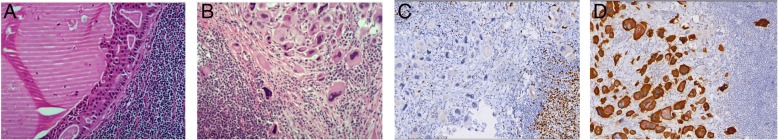
Lymph node metastases. (**a**) Cystic areas in the lymph node metastases (H&E, × 400). (**b**) Pleomorphic tumor giant cells in the lymph node metastases (H&E, × 400). (**c**) Negative reaction for Ki67 in pleomorphic tumor giant cells (Immunostaining, × 100). (**d**) Positive reaction for Her-2 in pleomorphic tumor giant cells (Immunostaining, × 100)

Microscopically, multiple variable-sized cystic spaces filled with thyroid colloid-like eosinophilic secretions [Fig. 2a] which was diastase resistant PAS positive and thyroglobulin negative. The eosinophilic secretions were retracted from the surrounding epithelia, producing scalloped margins. The cyst lining epithelium exhibited a variable pattern. In some areas the lining was flat to cuboidal epithelium and devoid of cellular atypia [Fig. 2b]. In other areas the epithelium showed a proliferative change in the form of pseudo stratification, knobby tufts [Fig. 2c], micropapillary [Fig. 2d] and cribriform [Fig. 2e]. An invasive component comprising of irregular neoplastic glands or nests was seen [Fig. 2f]. Eight axillary lymph nodes showed macro metastasis and cystic areas were also seen in the lymph node metastases [Fig. 3a]. Immunohistochemistry shows, the cystic contents were negative for thyroglobulin. Prognostic markers were ER negative, PR negative and HER2 3+. Ki67 was 30% positive. A diagnosis of invasive CHC with axillary node metastasis was made.

## Discussion

CHC is an uncommon subtype of duct carcinoma. The usual clinical presentation of CHC is a palpable lump and rarely nipple discharge. Calcification may be found in some cases by molybdenum target x-ray [8]. The present case had a long onset time (3 years). The lump was gradually progressive in size with an obvious skin ulceration. Microscopically, all features of cystic hypersecretory lesions were observed, including CHH, CHC, and focal invasive carcinoma. Eight axillary lymph nodes showed macro metastasis. Metastatic foci had cystic foci that contained eosinophilic secretion. These indicate us that though CHC of the breast behaves in a low-grade fashion for many years, but, nevertheless, has a potential for invasive growth and development of distant metastasis. It is interesting that some pleomorphic tumor giant cells were seen in the lymph node metastases. The nuclear morphometry of the giant cell was bizarre, while the nucleus to cytoplasm ratio was normal and without nuclear mitosis [Fig. 3b]. The morphology of these cells was resemblance to degenerative cells. Ki67 was negative [Fig. 3c] and Her-2 3+ [Fig. 3d]. The patient was not received chemotherapy or radiation therapy before surgery, so we think that these degenerative cells may be spontaneous alternation of the tumor cells in the metastases.

The differential diagnosis of invasive CHC includes ductal carcinoma in situ (DCIS) with comedo necrosis, secretory carcinoma, mucocele-like lesion, and metastatic thyroid carcinoma. 1. DCIS with comedo necrosis: Grossly, DCIS with comedo necrosis showed an ill-defined, yellow-white, granular appearance. Comedo necrosis can be seen in the cut surface. Microscopically, dilated ducts filled with necrotic material instead of thyroid colloid-like eosinophilic secretions. 2. Secretory carcinoma: Secretory carcinoma is also known as “juvenile secretory carcinoma”. It occurs most frequently in women of childbearing age with an average onset age of 25 years old. Secretory carcinoma contains a microcystic honeycomb appearance, eosinophilic secretion and vacuolar cytoplasm, while CHC is characterized by large dilated cystoid structures; 3. mucocele-like lesion: Grossly, mucocele-like lesion is similar to CHC. It also demonstrates cystically dilated ducts containing gelatinous or mucous material. Microscopically, secretions of the lesion are pale blue, basophilic and often accompanied by gross calcification, which are not typical features of CHC. 4. Metastatic thyroid carcinoma:Metastatic follicular thyroid cancer may resemble CHC. Histological differentiation of these two lesions may require immunohistochemical staining of thyroglobulin. Negative reaction for thyroglobulin was observed in the cyst contents of our case.

## Conclusion

Invasive cystic hypersecretory carcinoma is a rare subtype of breast cancer, characterized by the marked secretory activity of a thyroid colloid–like substance and cyst formation lined by pseudostratified to micropapillary epithelium along with foci of invasion. Owing to a smaller number of reported cases, little is known about the biological behavior, prognosis and molecular study of cystic hypersecretion lesions. Therefore, more cases with follow-up data are needed to reveal the biological behavior of this rare tumor.

## Abbreviations

CHC: Cystic Hypersecretory Carcinoma
CHH: Cystic Hypersecretory Hyperplasia
DCIS: Ductal Carcinoma in Situ
